# Supplementary material for: Identification of photoperiod-induced specific miRNAs in the adrenal glands of Sunite sheep (Ovis aries)
Source: Front Vet Sci. 2022 Jul 22;9:888207. doi: 10.3389/fvets.2022.888207 (PMC9354845; doi:10.3389/fvets.2022.888207)
Supplement: Supplementary file 1 [file Data_Sheet_1.ZIP › Supplementary file/miRDeep2 file.zip!/miRDeep2 file/Know_Structure/SPLP42A1/oar-mir-26b.pdf]

miRBase precursor : oar-mir-26b
Total read count : 160021
oar-miR-26b read count : 159886
remaining reads : 135

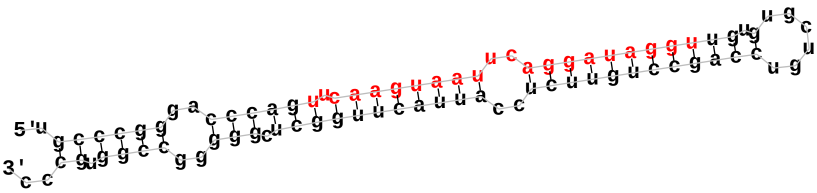

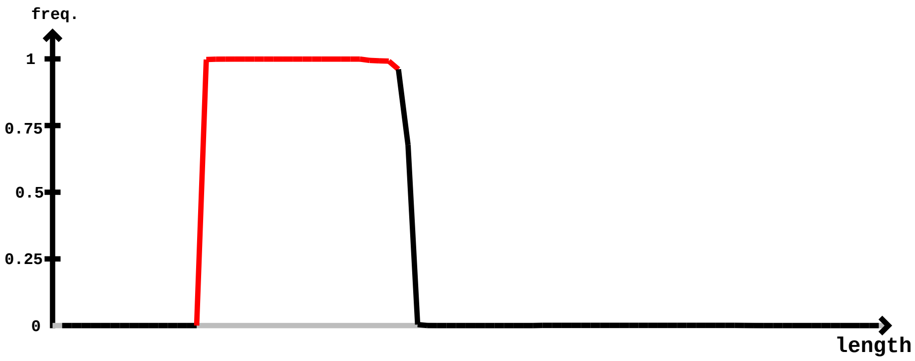


```
                  oar-miR-26b
5'- ugcccgggacccaguucaaguaauucaggauagguugugugcuguccagccuguucuccauuacuuggcucgggggccggugccc   -3'    exp
    .((((((..((((((.((((((((..(((((((((((.(......))))))))))))..))))))))))).))).)))).))..    reads      mm         sample
    .............guucaaguaauCcaggauag....................................................    1          1          seq
    .............guucaaguaauucaggauaggu..................................................    1          0          seq
    ..............uucaaguaauucaggau......................................................    594        0          seq
    ..............uCcaaguaauucaggau......................................................    3          1          seq
    ..............uucaaguaauucagUau......................................................    1          1          seq
    ..............uucaGguaauucaggau......................................................    3          1          seq
    ..............Aucaaguaauucaggau......................................................    2          1          seq
    ..............uucaaguaauucaggaC......................................................    80         1          seq
    ..............uucaaguaauucaggaA......................................................    19         1          seq
    ..............uucaaguaauucaggaG......................................................    19         1          seq
    ..............uuUaaguaauucaggau......................................................    4          1          seq
    ..............uucaaguaauuUaggau......................................................    1          1          seq
    ..............uucaUguaauucaggau......................................................    2          1          seq
    ..............uucaaguaaCucaggau......................................................    1          1          seq
    ..............uucaagCaauucaggau......................................................    4          1          seq
    ..............uucCaguaauucaggau......................................................    41         1          seq
    ..............uucaaguaauucaggaCa.....................................................    1          1          seq
    ..............uucaaguaauucaggaAa.....................................................    3          1          seq
    ..............uucCaguaauucaggaua.....................................................    18         1          seq
    ..............uucaaguaauucaggaua.....................................................    238        0          seq
    ..............uucaaguaauucaggauag....................................................    171        0          seq
    ..............uucaaguaauucaggauagg...................................................    4895       0          seq
    ..............uucaaguaauucaggauaggu..................................................    45380      0          seq
    ..............uucaaguaauucaggauagguu.................................................    107552     0          seq
    ..............uucaaguaauucaggauagguug................................................    568        0          seq
    ..............uucaaguaauucaggauagguugu...............................................    34         0          seq
    ..............uucaaguaauucaggauagguugug..............................................    1          0          seq
    ...............ucaaguaauucaggaua.....................................................    2          0          seq
    ...............ucaaguaauucaggauagg...................................................    10         0          seq
    ...............ucaaguaauucaggauaggu..................................................    55         0          seq
    ...............ucaaguaauucaggauagguu.................................................    132        0          seq
    ...............ucaaguaauucaggauagguug................................................    2          0          seq
    ...............ucaaguaauucaggauagguugu...............................................    1          0          seq
    ................caaguaauucaggauagg...................................................    1          0          seq
    ................caaguaauucaggauaggu..................................................    7          0          seq
```

oar-miR-26b

ugcccgggacccaguucaaguaauucaggauagguugugugcuguccagccuguucuccauuacuuggcucgggggccggugccc

................caaguaauucaggauagguu...	25	0	seq
.................aaguaauucaggauaggu..	2	0	seq
.................aaguaauucaggauagguu...	11	0	seq
..................aguaauucaggauagguu...	1	0	seq
....ccuguucuccauuacuugg.................	1	0	seq
....ccuguucuccauuacuuggc................	6	0	seq
....ccuguucuccauuacuuggcu...............	56	0	seq
....ccuguucuccauuacuuggcuc..............	49	0	seq
....ccuguucuccauuacuuggcucg.............	22	0	seq
....cuguucuccauuacuuggcucg.............	1	0	seq
